# Diagnostic performance of actigraphy in Alzheimer’s disease using a machine learning classifier – a cross-sectional memory clinic study

**DOI:** 10.1186/s13195-025-01751-5

**Published:** 2025-05-21

**Authors:** Mathias Holsey Gramkow, Andreas Brink-Kjær, Frederikke Kragh Clemmensen, Nikolai Sulkjær Sjælland, Gunhild Waldemar, Poul Jennum, Steen Gregers Hasselbalch, Kristian Steen Frederiksen

**Affiliations:** 1 Danish Dementia Research Centre, Department of Neurology, Copenhagen University Hospital - Rigshospitalet, Copenhagen, Denmark; 2 Department of Health Technology, Technical University of Denmark, Kongens Lyngby, Denmark; 3 Department of Clinical Neurophysiology, Danish Center for Sleep Medicine, Copenhagen University Hospital - Rigshospitalet, Copenhagen, Denmark; 4 Department of Clinical Medicine, Faculty of Health and Medical Sciences, University of Copenhagen, Copenhagen, Denmark; 5 Danish Dementia Research Centre, Department of Neurology, Copenhagen University Hospital – Rigshospitalet, Inge Lehmans Vej 8, Copenhagen, DK-2100 Denmark

**Keywords:** Actigraphy, Activity types, Physical activity, Alzheimer’s disease, Diagnosis, Digital biomarker

## Abstract

**Background:**

Movement patterns, activity levels and circadian rhythm are altered in Alzheimer’s disease (AD) and can be assessed by actigraphy using wearable sensors. We aimed to determine the diagnostic performance of actigraphy in AD in a memory clinic population by using a machine-learning classifier.

**Methods:**

In our single-center cross-sectional study, 70 patients with AD (MCI-moderate dementia), dementia with Lewy bodies (DLB) (*N* = 29) and cerebrovascular disease (CVD) (*N* = 23), and 48 elderly healthy controls were included. Participants underwent actigraphy at home using two body-worn sensors (SENS Motion^®^) for 1 week. We derived movement patterns (walking, running, resting, etc.) from raw accelerometry data using a proprietary algorithm. By evaluating the movement patterns during day and nighttime, we calculated 510 activity-related features, including robustness and fragmentation of the circadian rhythm. These features were used to train a machine learning (ML) classifier using logistic regression. We evaluated the performance of our classifier by assessing the accuracy and precision of predictions.

**Results:**

We found that movement patterns as well as the robustness and fragmentation of the circadian rhythm differed significantly between groups. During the daytime, patients with AD performed less moderate activity and walked less than the healthy group. While we achieved a modest accuracy of 68.8% for differentiating AD and healthy, the performance was highest (accuracy: 80−89%; precision: 69−84%) when ML was applied to actigraphy data to differentiate dementia etiologies (AD vs. DLB + AD vs. CVD).

**Conclusion:**

Actigraphy accurately identifies different dementia etiologies and could serve as a supplement to diagnostic investigations in patients with suspected AD for differential diagnostic purposes.

**Supplementary Information:**

The online version contains supplementary material available at 10.1186/s13195-025-01751-5.

## Introduction

Wearable digital health technologies represent a promising approach for assisting in the diagnosis of Alzheimer’s disease (AD) [1]. There is a large unmet need for easily applicable diagnostic markers, due to an expected increase in the prevalence of AD and recently approved disease-modifying therapies [2–4]. Actigraphy can capture physical activity levels in a home environment [5], has been used previously to assess circadian function and behavioral changes associated with AD [6–10], and thus may serve diagnostic purposes. As recently proposed [11], a paradigmatic shift is needed to accommodate the disease burden of AD. Digital health technologies, such as actigraphy, are expected to play a major role in this transition towards a digitized memory clinic and could be add-on diagnostic tools.

A major challenge for diagnosing AD correctly is the differential diagnoses versus other dementia disorders, such as dementia with Lewy bodies (DLB), and vascular cognitive dysfunction (VCD). Patients with DLB and VCD are frequently evaluated in memory clinics [12] and patients with DLB show a high degree of co-pathology with AD making a correct diagnosis difficult [13]. Tools to help differentiate these entities in a clinical setting are therefore needed. These tools should preferably be less costly and more easily accessible.

Activity levels exhibit a decline in patients with AD and dementia in general [8, 14], and increased fragmentation of the circadian rhythm has been shown already in the preclinical stages of AD [15, 16]. A number of behavioral changes, due to neuropsychiatric symptoms such as apathy, are prevalent at an early stage of the disease, which can be reflected in a lower overall activity level [6]. A larger cohort study from 2012 showed that decreased activity levels are associated with increased risk of developing AD and that lower activity levels were predictive of greater cognitive decline [17].Whether qualitative differences exist relating to which activity types (e.g., walking, running, cycling) are particularly affected is yet to be explored, although a recent study highlighted differences for moderate-intensity activity, being significantly lower in individuals with mild AD [18]. As actigraphy offers an inexpensive method of assessing these aspects and has shown promise in detecting salient features of AD [19], the technology may be used to assist in diagnosing AD. An additional aspect is the ability of actigraphy to capture natural behavior outside clinic settings. Traditional scales for assessing activities of daily living (ADL) are limited by the need for proxy rating [20]. As such, actigraphy may be envisioned as a method for capturing and tracking day-to-day variability as a digital biomarker for general ADL functioning.

Due to the multidimensionality of actigraphic data, hypothesis-driven approaches, while valid for certain research questions, may overlook important elements of time-distributed complex data [21]. As such, machine learning provides a method for selecting relevant parameters in predictive modeling, without this pitfall. In the present study, we investigated a machine learning approach for predicting an AD diagnosis in a well-described memory clinic cohort, using actigraphy-derived activity categories and circadian rhythm with appropriate comparators, namely aged healthy controls, patients with DLB, and patients with cerebrovascular disease. We hypothesized that actigraphy could differentiate healthy and disease states, and that actigraphy had differential diagnostic potential.

## Materials and methods

### Study design

Single-center, cross-sectional diagnostic study.

### Participants

**Fig. 1:**
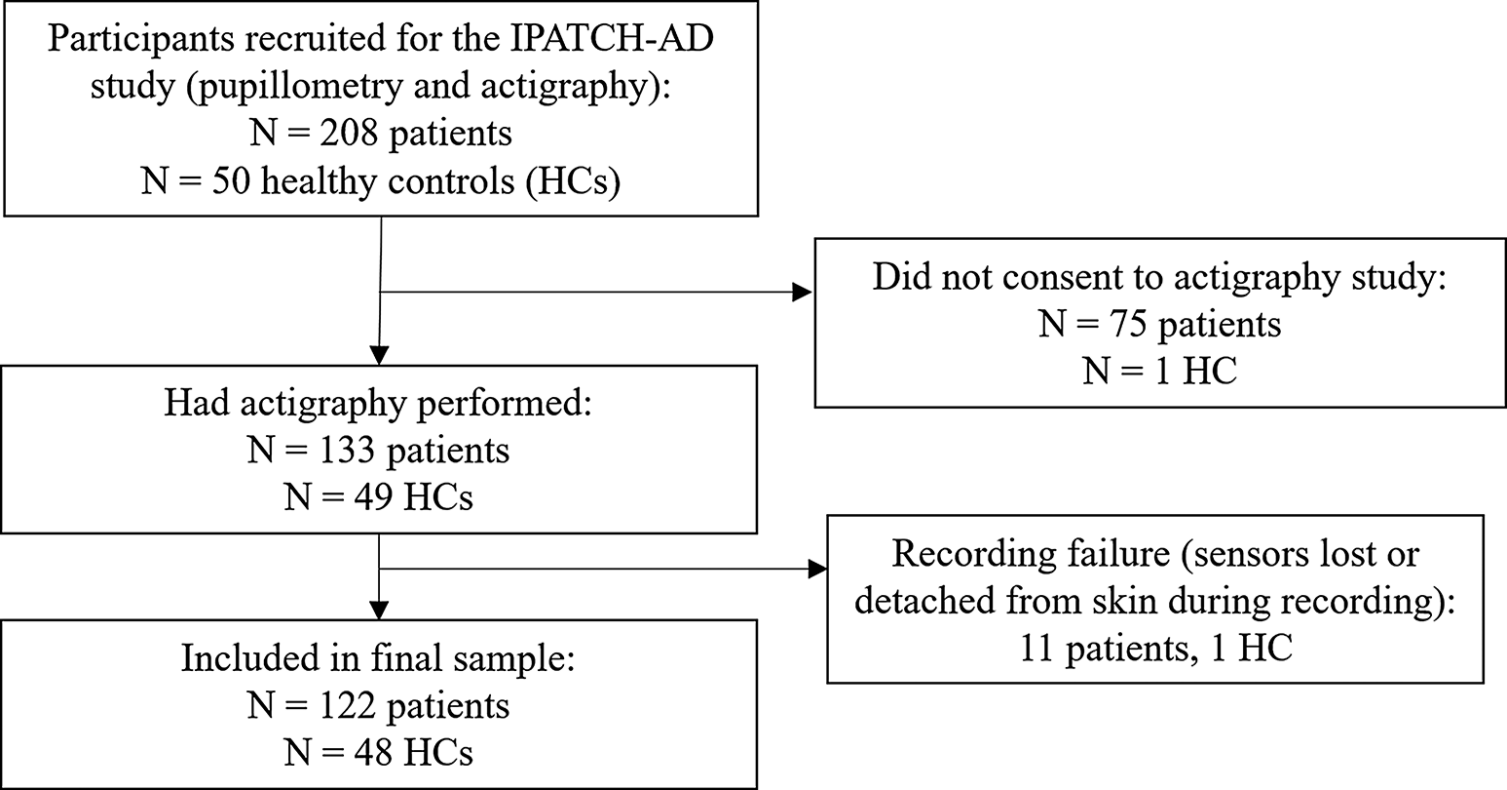
Flow chart for the actigraphy part of the IPATCH-AD study

This study is part of an investigation of digital health technologies, from which results on quantitative light reflex pupillometry have been reported [22]. The recruitment procedure has been described previously, and the inclusion of participants in the present study is shown in a flowchart in Fig. 1. Briefly, patients were diagnosed at a university memory clinic and underwent comprehensive diagnostic assessment. The diagnosis was given following a multi-disciplinary (neurology, geriatrics, psychiatry, neuropsychology, nurses) consensus conference.

Patients were included if they fulfilled the inclusion criteria: (1) a diagnosis of AD (both mild cognitive impairment (MCI) and dementia stages) [23, 24], AD with mixed pathology (cerebrovascular co-pathology) [24], cognitive dysfunction due to cerebrovascular disease [25], or dementia with Lewy bodies (MCI and dementia) [26, 27], (2) An MMSE total score > 15, (3) able to provide informed consent and none of the following exclusion criteria: (1) diagnosis of concurrent neurological (other than AD) or psychiatric disorder (mild depression allowed), (2) excessive alcohol intake > 5 units per day or substance use, (3) concurrent participation in interventional studies, (4) other known brain disorder which could cause cognitive dysfunction. Healthy controls were included if they fulfilled the following inclusion criteria (1) ability to cooperate with the investigations, (2) normal cognitive function as evaluated by the investigator, (3) age between 50 and 90 years, and none of the listed exclusion criteria applied to patients. Patients were not institutionalized, except for one patient with DLB.

The study (IPATCH-AD) was registered at clinicaltrials.gov (Clinical Trial No.: NCT05175664, registration date: 4th of January 2022) and carried out with permission from the regional ethics committee (file number: H-21045068). The tenets of the 1975 Helsinki Declaration were followed, and written informed consent was obtained from all participants ensuring the capacity to provide consent after consulting the memory clinic physician responsible for the patient.

### Cognitive testing and demographic data

The Mini-Mental State Examination (MMSE) [28], assessing global cognitive function, was administered by the investigator for the healthy controls. MMSE score, administered by a doctor at the most recent visit to the memory clinic was used for patients. Demographic data, including data on age, sex, educational level, and information on medication use that could influence the circadian rhythm (antidepressants, antipsychotics, hypnotics and sedatives, and anxiolytics), was extracted from the medical files and entered into an electronic database (REDCap, Vancouver). Healthy controls filled out a questionnaire, where this was addressed as well. Patients and controls were also asked to gauge their maximal walking distance in a single session (answers were capped at 10.000 m) as a measure of functional capacity.

### Procedure

#### Actigraphy

Patients and controls had actigraphy performed in their home environment using the SENS® Motion Actigraph System for a full 7-day week as an opt-in part of IPATCH-AD (NCT05175664) (for flow chart, see Fig. 1). The system consists of an accelerometer with a 12.5 Hz sampling rate that measures acceleration (-4-+4 *g*) in the *xyz*-coordinate system, a chronometer and a thermometer embedded in a 47 × 22 × 4.5 mm large unit incorporated in a medical adhesive patch (3M®). One sensor was applied on the sternum or below the mid part of the clavicula, to indicate upper body position, and one sensor was applied on the lateral thigh (10 cm proximal to the lateral femoral epichondylum) of the participant to capture movement.

#### Pre-processing

The SENS Motion system uses a proprietary algorithm for classifying activity, evaluated for each 5-second epoch. This algorithm has previously been shown to have high concordance with validated movement behavior in elderly patients [29]. The activity categories are (1) upright standing, (2) sporadic walking, 2) walking, 4) running (high intensity), 5) moderate intensity (brisk walking) 6) lying rest, 7) lying movement, 8) sitting. The exact definition of each activity category is described in the Supplementary Material.

**Fig. 2:**
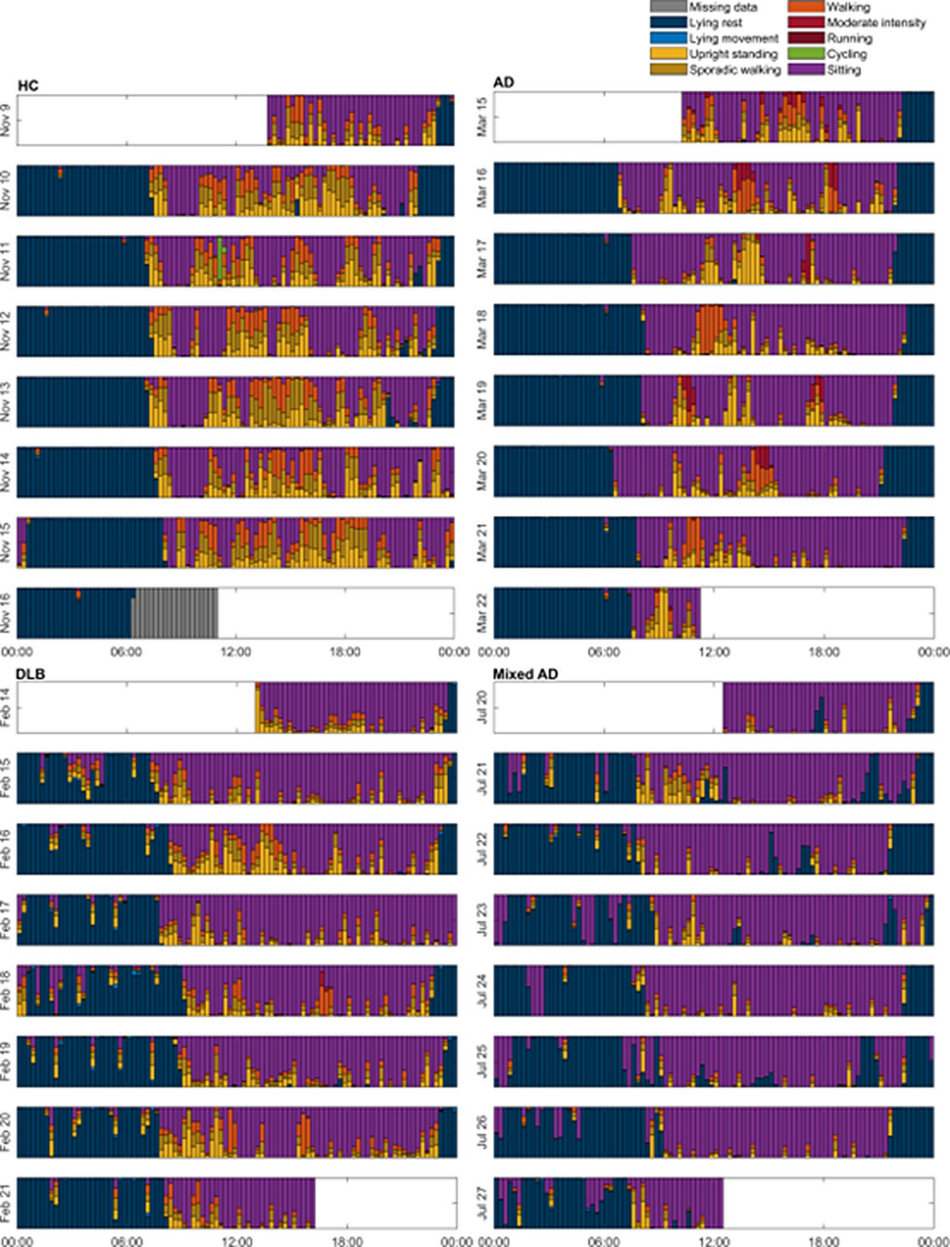
Actigram from a healthy control (HC), a patient with Alzheimer’s disease (AD), dementia with Lewy bodies (DLB), and mixed AD (+ cerebrovascular pathology) showing the distribution of activity patterns during a 7-day wear period

The amount of each activity category over a 7-day wear week from which an actigram can be constructed depicting a daily activity pattern is illustrated in Fig. 2.

#### Feature extraction

The SENS software provides activity categories and activity count measures in 5.128-second, 15-minute, and 1-hour windows. The 15-minute resolution was chosen as a compromise between temporal resolution and variability of data.

These measures were subsequently analyzed in three time windows (24-hour period: 10 PM – 10 PM, night: 12 midnight − 6 AM, day: 9 AM – 9 PM). In these periods, the following features were calculated for each of the activity categories: (a) mean, (b) percentage of windows with above $\:x\in\:\{5,\:10,\:25\:\text{\%}\}$ of time spent in activity category, (c) intra-daily variability:

1 $$\:\begin{array}{c}IV=\frac{n\cdot\:\sum\:_{i=2}^{n}{\left({x}_{i}-{x}_{i-1}\right)}^{2}}{\left(n-1\right)\cdot\:\sum\:_{i=1}^{n}{\left(\stackrel{-}{x}-{x}_{i}\right)}^{2}}\end{array}$$

where $\:x$ is the activity categories, $\:\stackrel{-}{x}$ is the average of $\:x$, and $\:n$ is the length of $\:x$. The activity count measures were summarized using (a) mean, (b) mean during sitting activity category, (c) activity during the 10 most active hours (+ timing), (d) activity during the 5 least active hours (+ timing), (e) relative amplitude: (M10 - L5) / (M10 + L5), (f) intra-daily variability (Eq. 1). In total, this adds up to 255 features that were computed for each 24-hour period.

To compute summary statistics, these were summarized using average and variance for all available 24-hour periods per subject resulting in 510 features. Moreover, for activity count measures, the average curve of each 24-hour cycle was computed for each diagnostic group. Summary statistics (mean and standard deviation) for all calculated features for each diagnostic group are available in the Supplementary Material.

#### Machine learning

Machine learning was carried out using logistic regression combined with sequential feature selection filtered by a minimum redundancy maximum relevance algorithm. All models were fitted and evaluated in a nested leave-one out cross-validation setup, and selected features were evaluated in the inner loop. No further regularization was used. As a comparison, a model using a single feature, the average intensity count during the 24-hour period was also used. As the mixed AD and vascular cognitive dysfunction groups were similar on several parameters, these were combined for the machine learning analyses. The evaluation metrics were sensitivity, specificity, accuracy, precision and the F1 score:

$$\:F1=2\cdot\:\frac{\text{precision}\cdot\text{sensitivity}}{\text{precision}+\:\text{sensitivity}}$$

The F1-score, which is the harmonic mean of precision and sensitivity, is commonly used to assess the performance of machine learning models. It penalizes extreme measures of either precision or sensitivity [30]. Feature importance was evaluated by examining the average absolute values of logistic regression coefficients.

### Statistical analysis

Python, R (ver. 4.2.2) and MATLAB (R2019b, The MathWorks, Natick, Massachusetts, USA) were used for all analyses.

Quantitative variables are expressed by their mean and standard deviation. Variables were assessed for normality by visually inspecting histograms. Parametric and non-parametric tests were used to test group differences as appropriate. As actigraphic parameters exhibited non-normal distribution, a Kruskal-Wallis rank sum test was used to evaluate group differences. Performance confidence intervals were estimated using Clopper–Pearson’s method. Performance for the logistic regression was evaluated at the default thresholds and at an optimized threshold by finding the point closest to (0,1) on the ROC curve. We imposed an alpha level of 0.05 and used only two-tailed tests.

## Results

As shown in Table 1, a total of 122 patients and 48 healthy controls were included. There were significant differences between diagnostic groups for age, sex, and educational level, while no significant group differences were found for the use of antidepressant, antipsychotic, hypnotic, or anxiolytic medication (Table 1). Disease groups were older than the control group, and a higher proportion of patients with DLB were male. The majority of patients across disease groups had MCI or mild dementia. MMSE total scores were lower for dementia groups and lowest in the mixed dementia group. There were more patients with DLB or vascular cognitive dysfunction (mixed AD and pure vascular dementia) who had objective gait impairment. Likewise, the subjective walking distance was lower in dementia groups, with the lowest found for the mixed dementia group.

**Table 1:** Cohort characteristics

	Alzheimer’s disease (*N* = 70)	Dementia with Lewy bodies (*N* = 29)	Mixed AD (+ CVD) (*N* = 8)	Vascular cognitive dysfunction (*N* = 15)	Healthy control (*N* = 48)	*P*-value
**Age (years**), mean (SD)	75.3 (7.1)	76.2 (6.4)	81.8 (3.3)	78.5 (5.9)	71.1 (7.8)	< 0.001^1^
**Sex**, n (%)						
Female	33 (47.1%)	4 (13.8%)	4 (50.0%)	5 (33.3%)	30 (62.5%)	< 0.001^2^
Male	37 (52.9%)	25 (86.2%)	4 (50.0%)	10 (66.7%)	18 (37.5%)	
**MMSE total score**, mean (SD)	25.5 (2.7)	26.2 (2.9)	22.6 (2.8)	26.5 (2.9)	29.1 (1.1)	< 0.001^3^
**Educational level (years)**, mean (SD)	11.8 (3.7)	13 (4)	13.3 (4.7)	10.5 (2.9)	15 (2.5)	< 0.001^3^
**Disease severity**, n(%)						
Mild cognitive impairment	21 (30.0%)	3 (10.3%)	0 (0.0%)	8 (53.3%)	-	< 0.001^2^
Mild dementia	46 (65.7%)	25 (86.2%)	5 (62.5%)	4 (26.7%)	-	
Moderate dementia	3 (4.3%)	1 (3.4%)	3 (37.5%)	3 (20.0%)	-	
**Objective gait impairment**, n (%)						
No	63 (90.0%)	11 (37.9%)	4 (50.0%)	8 (53.3%)	45 (93.8%)	< 0.001^2^
Yes	7 (10.0%)	17 (58.6%)	4 (50.0%)	6 (40.0%)	3 (6.2%)	
**Subjective maximum walking distance (meters)**, mean (SD)	6137 (3205)	4598 (3473)	1775 (1639)	3314 (3303)	7523 (3144)	< 0.001^3^
**Hypnotic medication**, n (%)						
Yes	1 (1.4%)	0 (0.0%)	0 (0.0%)	0 (0.0%)	0 (0.0%)	0.697^2^
**Previous stroke**, n (%)						
Yes	2 (2.9%)	1 (3.4%)	2 (25.0%)	5 (33.3%)	0 (0.0%)	< 0.001^2^
**Sedatives or analgesic medication**, n (%)						
Yes	1 (1.4%)	2 (6.9%)	0 (0.0%)	0 (0.0%)	0 (0.0%)	0.126^2^
**Antidepressant medicine**, n (%)						
Yes	6 (8.6%)	4 (13.8%)	1 (12.5%)	3 (20.0%)	3 (6.2%)	0.429^2^
**Antipsychotic medication**, n (%)						
Yes	1 (1.4%)	1 (3.4%)	0 (0.0%)	1 (6.7%)	0 (0.0%)	0.519^2^
**Antidementia medication**, n (%)						
Yes	29 (41.4%)	22 (75.9%)	6 (75.0%)	0 (0.0%)	0 (0.0%)	< 0.001^2^

All variables had < 5 missing values. Frequency is calculated for participants without missing data

^1^ANOVA

^2^Fisher’s Exact Test

^3^Kruskal-Wallis rank sum test

**Fig. 3:**
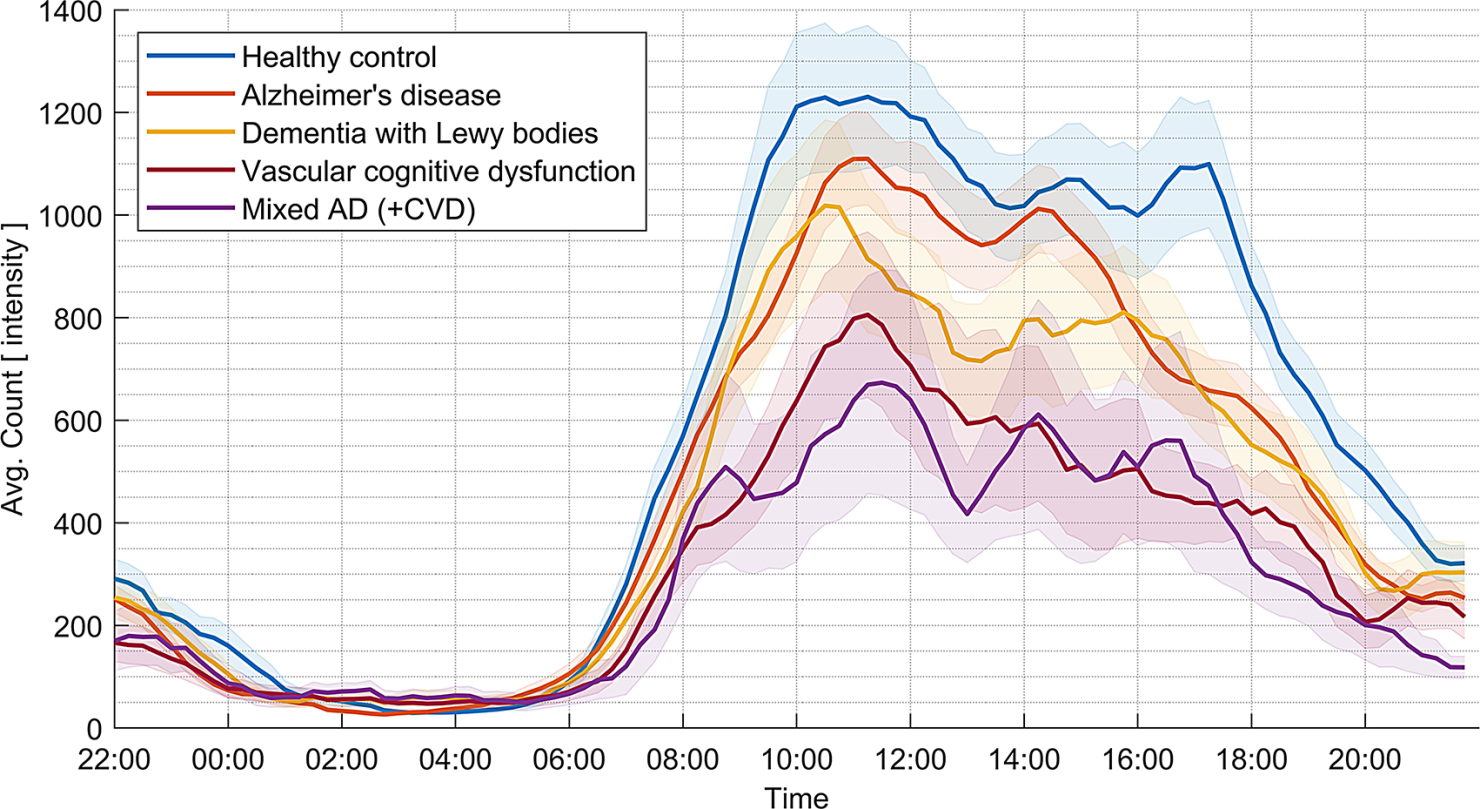
Average intensity count during the 24-hour period for each diagnostic group. The curve is based on the 15-minute resolution and smoothed within the closest hour. Shaded areas represent the standard error of the mean

**Fig. 4:**
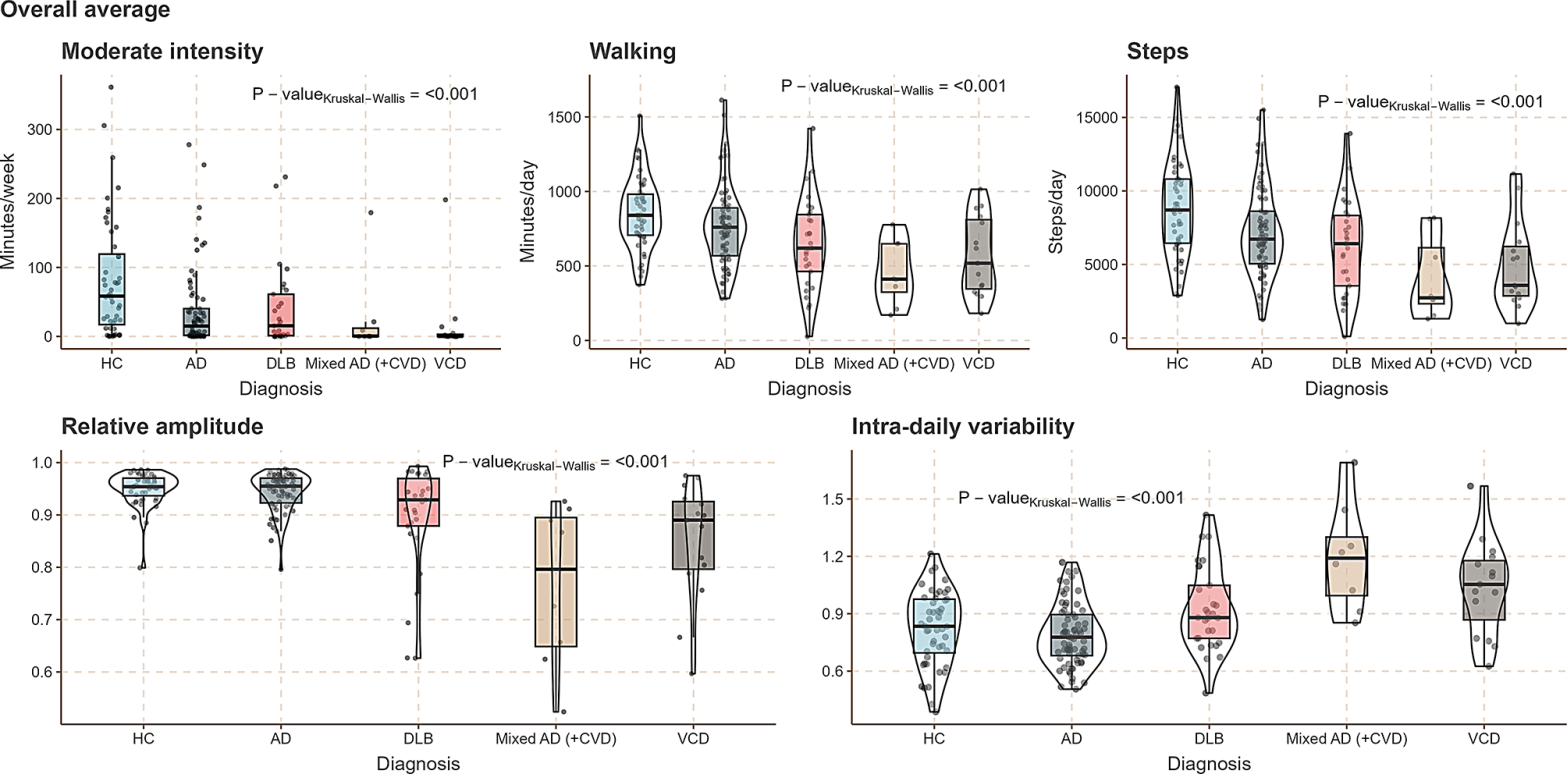
Box- violin-, and dotplots showing the proportion of time spent in activity categories and circadian indices for all groups. Boxplots show the median and inter-quartile ranges (IQR), whiskers are 1,5 x IQR. HC = healthy control, AD = Alzheimer’s disease, DLB = dementia with Lewy bodies, Mixed AD (+ CVD) = mixed Alzheimer’s disease (+ cerebrovascular disease), VCD = vascular cognitive dysfunction. P-values represent between-group differences

When we examined the overall proportion of the time spent performing each activity type there were significant between-group differences for the lying rest category, which was numerically slightly higher in disease groups (Table 2). When examining average intensity counts, as an overall measure of all activity types, these were highest for healthy controls (Fig. 3). Patients with dementia exhibited less walking activity, with the most pronounced differences between healthy controls and mixed dementia (Fig. 4). Significant between-group differences were found for relative amplitude reflecting the stability of the circadian rhythm, with disease groups showing a less robust circadian rhythm, although AD and healthy controls were similar on this parameter. A similar pattern was found for the intra-daily variability, indicating fragmentation of circadian rhythm, with a numerically more fragmented rhythm for disease groups, which again was less pronounced for the AD group. Averaging activity counts throughout the measurement period for each participant category revealed overall lower activity levels throughout the day with the lowest activity level for the mixed dementia group (Fig. 3).

**Table 2:** The mean overall time spent in activity categories, the activity level and circadian indices for each diagnostic group

	Alzheimer’s disease (*n* = 70)	Dementia with Lewy bodies (*n* = 29)	Mixed AD (+ CVD) (*n* = 8)	Vascular cognitive dysfunction (*n* = 15)	Healthy control (*n* = 48)	*P*-value
**Activity categories**						
**Lying rest**, minutes/day	591 ± 102.7	625.4 ± 119.2	568.9 ± 101	633.1 ± 107.3	539.7 ± 88.8	**0.005**
**Lying movement**, minutes/day	5.7 ± 3.8	5.4 ± 3.6	6.4 ± 3.2	5.1 ± 3.4	6.3 ± 3.3	0.3
**Upright standing**, minutes/day	120 ± 53.6	116.8 ± 48.7	145.3 ± 65.3	89 ± 42.6	117.3 ± 40.1	0.05
**Sporadic walking**, minutes/day	116.3 ± 40	98.5 ± 37.3	89.4 ± 35.4	82.1 ± 52.2	108.1 ± 32.1	**0.002**
**Running**, minutes/week	3.1 ± 20.7	4.4 ± 15.5	0.3 ± 0.6	0.1 ± 0.3	3.8 ± 10.4	**0.047**
**Cycling**, minutes/week	17.6 ± 40.6	60.3 ± 99.8	1.8 ± 5.1	11.2 ± 18.5	42.6 ± 65.9	**0.001**
**Sitting**, minutes/day	489.8 ± 112.8	486.4 ± 110.3	560.9 ± 151.4	545.6 ± 138	528.2 ± 96.3	**0.01**
**Sit-to-stand**, number/day	61 ± 19.7	65 ± 25.1	50 ± 21.8	52 ± 13.9	62 ± 15.8	0.06
**Activity level and circadian indices**						
**Overall intensity**, intensity count	31.7 ± 10.6	29.1 ± 13.8	19.5 ± 10.6	21.4 ± 11.2	39.1 ± 12.2	**< 0.001**
**Most active 10 h**, intensity count	926 ± 331	832 ± 414.9	548.6 ± 356.7	602.0 ± 353.4	1135.4 ± 401.8	**< 0.001**
**Least active five hours**, intensity count	22.2 ± 13.6	36.0 ± 30.4	43.5 ± 11.3	34.9 ± 25.5	24.5 ± 12.7	**0.002**

Values are expressed as the mean ± standard deviation and are given as average number of minutes per day or week spent in an activity category as classified by the algorithm. Intensity counts are unitless. P-values from Kruskal-Wallis test. **Bold** indicates p-value below significance level

The subjective maximal walking distance, an analog measure of functional capacity, followed activity levels closely (Table 1).

An in-depth examination of nighttime and daytime distribution of activity categories revealed only slight differences between groups (Supplementary Tables 1 and 2).

### Diagnostic machine learning classifier

#### AD vs. healthy controls

**Fig. 5:**
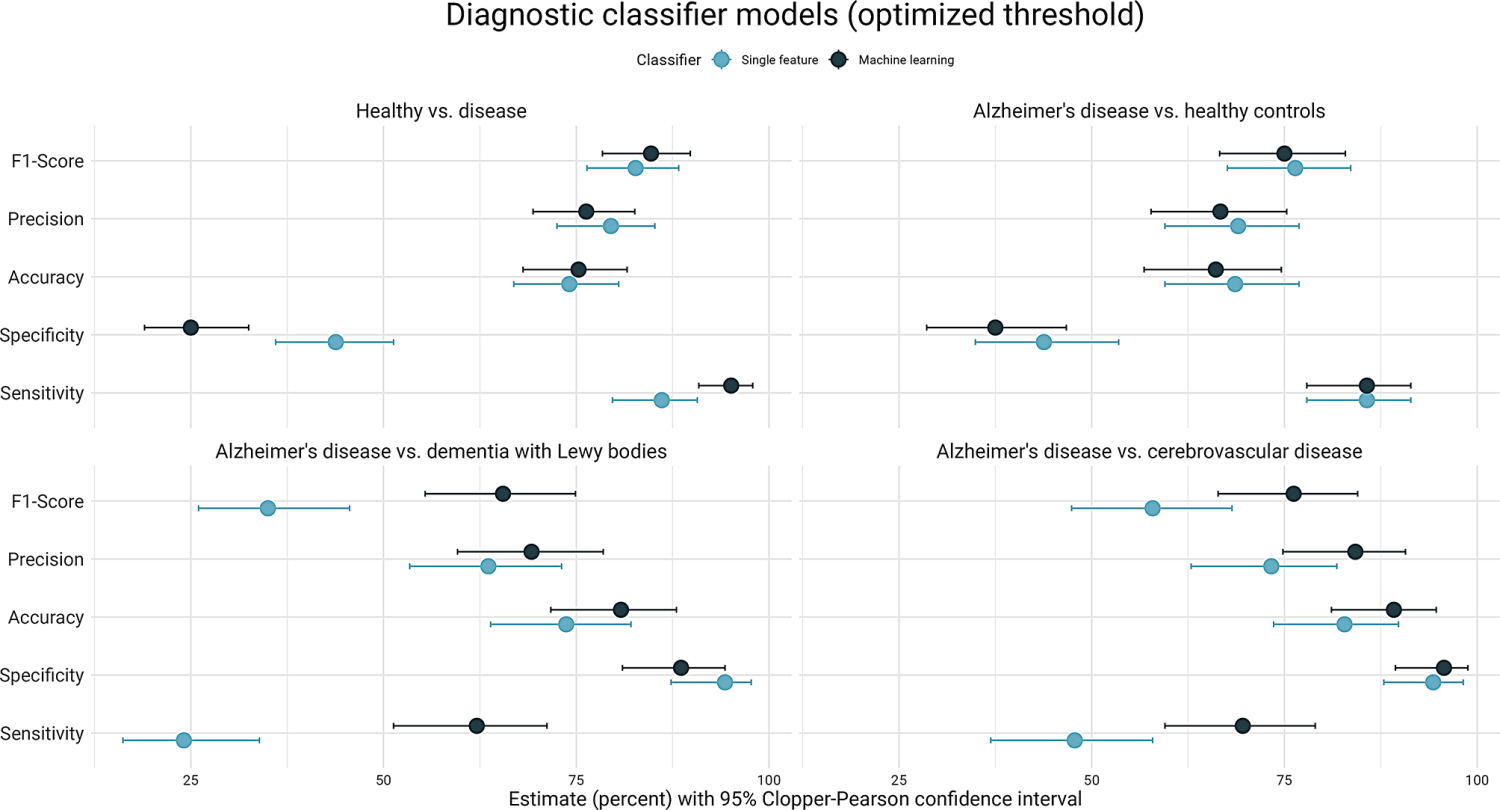
Performance in differentiating groups in a leave-one-out scheme using either logistic regression (machine learning) or thresholding of average intensity count during the day (single feature). The probability threshold was optimized by finding the point closest to (0,1) on the ROC curve

When distinguishing between AD and healthy controls, the machine learning classifier, which used all 510 features, performed similarly to the single feature classifier using only the overall daily activity levels (accuracy: 66.1% vs. 68.8%, F1: 75% vs. 76.4%) (Fig. 5 and Supplementary Table 3). Both achieved an F1-score above 75%. Sensitivity was similar for both models (85.7%) with slightly higher specificity for the single feature model (43.8%) when optimizing the discriminatory threshold, although the confidence intervals overlapped.

#### Healthy controls vs. disease groups

When we examined the predictive ability of actigraphy to distinguish between all disease groups (AD, DLB, CVD) and healthy controls, the model performed with an accuracy of 75.3%, a precision of 76.3%, and Dead an F1-score of 84.7% (95% CI: 78.4–89.8) for the optimized threshold (Fig. 5 and Supplementary Table 3). Compared to the single feature classifier, sensitivity was higher (95.1% vs. 86.1%), while the other performance measures (accuracy, precision and F1-score) indicated no additional information when choosing from the full set of features (Fig. 5 and Supplementary Table 3).

#### AD vs. DLB

The machine learning classifier outperformed the single feature classifier on all performance measures, except for specificity, achieving > 80% accuracy in predicting AD vs. DLB using all available actigraphy features. The F1-score also indicated a better overall performance for the machine learning model, maybe due to the substantially higher sensitivity (62.1% vs. 24.1%) (Fig. 5 and Supplementary Table 4).

#### AD vs. mixed AD (+ vascular pathology) and VCD

The full-feature set machine learning model, which had a sensitivity of 69.6% and specificity of 95.7%, outperformed the single feature model on sensitivity and achieved an accuracy of 89% with an F1-score of 76.2%, for which the confidence intervals overlapped with that of the single feature classifier (Fig. 5 and Supplementary Table 4). Performance for the default threshold classifier is shown in Supplementary Fig. 1.

#### Feature importance

Feature importance plots for all machine learning classifiers are shown in Supplementary Fig. 2. The absolute regression coefficients were numerically high for the healthy vs. disease classification. This indicated that the machine learning model may have experienced overfitting or little discriminatory value in the variables, as was also shown in the forest plot. Nonetheless, nighttime activity was one of the most important features for classification. For the DLB vs. AD ML classifier, the robustness of the number of steps, as a proxy for activity, was most important, and the distribution for lying rest showed high importance as well for this model. For the vascular vs. AD classification, the distribution and variability of walking activity as well as nighttime sitting activity were indicative of the disease class.

#### Output probability

We modeled the output probability of or ML models for healthy and dementia groups, separately, to test for possible confounders (age, sex, medication). The results of this analysis are shown in Supplementary Tables 5 and 6. This showed no concern for confounding in the control group (no significant predictors), while in the dementia group, age and the use of sedatives or analgesic medication had a significant effect on the output probability, indicating possible confounding, although the estimates were small.

## Discussion

We have evaluated the diagnostic performance of actigraphy coupled with an activity-type algorithm using machine learning to differentiate AD from aged, healthy controls and relevant disease comparators in a well-described memory clinic cohort. We generally found the highest diagnostic predictive ability with our machine learning approach, when the classifier was applied to differentiate between dementia etiologies, whereas this approach did not add to the differentiating ability when comparing overall disease groups or AD with healthy subjects. We found indications that the circadian rhythm was less robust and more fragmented in dementia groups when compared to aged, healthy controls, although the AD group was similar to the healthy group. The circadian phase was preserved across groups.

Interestingly, we found that combining actigraphy with a machine learning approach was most advantageous for differentiating dementia etiologies, as this approach achieved significantly higher sensitivity and higher accuracy when distinguishing both AD vs. DLB and AD vs. CVD compared to only using the overall daily mean activity level (single feature). To our knowledge, no other studies have evaluated actigraphically derived activity types for AD diagnosis. Utilizing the full scope of data, including nighttime periods, the machine learning approach performed seemingly better than single-feature classification, indicating that movement/activity patterns and subtle nighttime differences between groups might have improved the differentiation of etiologies. This was reflected by the most important features being fragmentation of walking activity, maybe indicating shorter walking bouts for the CVD group, and sitting activity during nighttime. The fragmentation of walking activity fits well with the literature describing a decrease in walking activity in stroke survivors [31], although the time distribution of walking has not been investigated previously. Although the existing literature is sparse on the comparison of AD to other dementias with actigraphy, a previous study reported no differences between AD and multi-infarct dementia [32], a finding we could not replicate, as we found that actigraphy was able to differentiate these etiologies with high accuracy. This may be explained by differences in our analysis method and two-sensor setup with a higher sampling frequency. Specifically, we applied an activity category algorithm, further classifying the movement patterns associated with each dementia etiology. Also, as diagnostic criteria for vascular cognitive dysfunction and AD have changed since the publication by Mishima et al., this may be reflected in the differences observed. Other groups have found associations between hallmarks of vascular cognitive dysfunction, such as higher levels of white matter hyperintensities and small vessel disease, and lower activity levels, which is in accordance with our results [33, 34].

The neuropathological underpinnings of a disturbed circadian rhythm, possibly also affecting activity levels, are mainly situated in the suprachiasmatic nucleus, where a lower number of neurons has been shown in patients with AD [35, 36]. Also, a recent study showed that hypopigmentation of the locus coeruleus, a key modifier of circadian function, was associated with cortical AD pathology at autopsy [37]. While we found lower activity levels in the AD groups relative to controls, we could not confirm the presence of a distinct circadian rhythm dysfunction in AD, which has frequently been reported in clinical [38–40] and pre-clinical stages [41], although not consistently [42]. Rather, patients with DLB and CVD exhibited a less robust and more fragmented circadian rhythm, which may have increased the discriminatory power of our models when differentiating dementia etiologies. Indeed, circadian fragmentation and short bouts of lying rest were one of the most important features in our models. While sleep disturbances are well-described in DLB, such as REM sleep behavior disorder, which is part of the diagnostic criteria for the disease [26], a distinct circadian dysfunction has not been characterized in larger studies [43]. Surprisingly, we did not detect differences in nighttime behavior as groups were largely similar during this period of recording. This suggests that thigh-worn actigraphy may not capture salient nighttime pathology in DLB, where for example RBD is highly prevalent [43]. To this end, wrist-worn actigraphy represents a more promising approach [21], which is also supported by the finding that REM sleep without atonia is more pronounced in the arms in comparison to the chin and legs [44]. In our study, we did not extract sleep metrics from the thigh-worn actigraphy due to limitations in the used algorithm. While we did not extract sleep metrics, our analysis of activity in the nighttime window may serve as a proxy for sleep-related behavior, although we cannot provide validation for this aspect. Future studies should evaluate sleep metrics alongside activity patterns to fully elucidate the discriminatory ability of thigh-worn actigraphy. Literature on the direct comparison of AD and DLB on actigraphic parameters measured in clinically diagnosed DLB is scarce, although post-mortem determined Lewy body co-pathology in AD patients has shown to be associated with circadian dysfunction [45]. Only one smaller study has looked at the discriminatory power of actigraphy in the distinction between AD and DLB [46], and our results are in accordance with these findings, namely a larger degree of variability in nighttime to daytime activity reflecting less robustness of circadian rhythm in the DLB group, as was also reflected by the robustness of number of steps being the top feature for AD vs. DLB classification. As co-pathology with AD, especially cerebral deposition of amyloid, is prevalent in DLB [47] it is crucial to develop tools, besides fluid biomarkers, that can aid in discriminating these etiologies in the memory clinic. To this end, actigraphy may represent a promising approach, and coupling this approach with machine learning seems useful. As such, our results provide a proof-of-concept for the application of actigraphy in a memory clinic setting for differential diagnostic purposes.While we investigated the diagnostic capability of actigraphy at the cross-sectional level, there is also an avenue for assessing activity-related changes as a proxy for disease progression in longitudinal design. These analyses are planned for a follow-up sub-cohort of the present study (ClinicalTrials.gov ID NCT05175664).

Groups were mostly similar regarding medication use that could influence the circadian rhythm and activity levels, but significant differences were found for subjective walking distance and gait impairment. This means that some differences in actigraphic parameters could have been driven by impaired functional capacity in certain diagnostic groups and important diagnostic information can thus be surveyed by asking simple questions about subjective walking distance, although the study was not designed to investigate this aspect. The DLB and CVD groups were older than healthy controls and the AD group meaning that age-related functional decline and circadian changes [41] could have confounded the results for comparisons with these groups. This was also indicated by investigating output probability of our ML model, showing that age was a significant predictor in the dementia groups. We also did not find a significant circadian phase shift, meaning that patients kept the circadian rhythm intact, possibly by aligning with known entrainers (light, social activity, temperature, etc.) [48, 49].

We found slightly lower overall activity levels in AD compared to healthy controls, possibly relating to less time spent walking and doing moderate-intensity activities (e.g., brisk walking), which was also found in a recent study [18], and more time spent resting for AD patients. The higher mean overall activity levels detected in the healthy controls could be due to a sustained afternoon activity level, as healthy controls showed an activity peak in the late afternoon, a pattern that was absent in dementia groups. Although the mean overall activity levels as detected by the actigraph were able to differentiate the groups with some certainty, a machine learning approach may not be useful for the distinction of AD and healthy controls.

As such, the application of actigraphy in a memory clinic setting seems viable for differential diagnostic purposes: the technology can be applied early in the diagnostic process and could weave seamlessly into the clinic as an add-on to traditional biomarkers (cognitive testing, cerebrospinal fluid samping, and neuroimaging) as it provides information on the natural behavior and circadian rhythm outside the clinic. In addition, it is associated with low costs and a low patient burden, which is preferred due to an expected increase in referrals to memory clinics in part due to expected demographic changes with increasing cases of dementia and due to recently approved disease-modifying therapies for AD [20, 50]. Together, this makes actigraphy an interesting digital tool to be explored further in memory clinic settings; this could be for disease-tracking, remote monitoring of treatment response, or early screening purposes, which are beyond the scope of this paper. However, the application of a ML approach should warrant caution regarding how results are communicated to patients, given that the algorithms applied are not always easily interpretable. With our chosen ML model, feature importance can be examined, while models such as neural networks are less easy to interpret [51]. Improving interpretability of ML models is an ongoing task and one that will need special refinement when it comes to informing patients with cognitive impairment.

The strengths of our study are the large, well-described cohort of patients and the relevant setting of the memory clinic, where using actigraphs with a high sampling rate could be envisioned as part of a diagnostic work-up. Further, we applied machine learning, utilizing the full scope of data rather than focusing on single variables, and ensured a qualitative assessment of activity levels by applying an activity-type algorithm from two body-worn sensors. All but one patient were non-institutionalized, meaning that our results reflected the natural behavior of participants, which increases the generalizability of our findings.

Limitations also apply to our study. First, we applied a pragmatic definition of day- and nighttime periods, which may have influenced our results, however, changing the periods slightly did not alter our results. Second, our choice of sensor placement, namely thigh and sternum, could have hindered the detection of subtle nighttime motor activity reflecting REM sleep behavior disorder, which is prevalent in DLB, and may be more easily detected using wrist-worn actigraphy. On the other hand, the chosen sensor placement was essential for correct activity category classification and for accurately determining physical activity levels. Third, the study lacked external validation in an independent cohort, preferably patients evaluated before diagnosis, however, we provide unbiased estimates from cross-validated analyses to adjudicate this. Fourth, relevant differential diagnoses such as MCI of unknown etiology or depression were not included. Fifth, the diagnostic groups were not balanced on demographics, which could have influenced our results, as age was a significant predictor of the output probability, albeit only for the dementia group. Sixth, we did not evaluate the added value of actigraphy to traditional biomarkers (e.g., cerebrospinal fluid, EEG, neuroimaging), however, assessing the stand-alone value of a novel biomarker seems crucial before conducting an add-on study design.

We have explored the clinical applicability of actigraphy in a memory clinic setting using a machine-learning approach. We found several actigraphic parameters that were altered in the dementia stage in accordance with recent literature. While our machine learning approach was less robust in differentiating healthy from disease, we did find that actigraphy was able to differentiate dementia etiologies with high accuracy, although part of the performance may have been driven by subtle age-differences. Actigraphy could therefore potentially be used for differential diagnostic purposes in memory clinics, provided age differences are taken into account. Future studies should aim to validate these findings in other memory clinic cohorts and evaluate actigraphy alongside traditional biomarkers.

## Electronic supplementary material

Below is the link to the electronic supplementary material.

Supplementary Material 1

Supplementary Material 2

## Data Availability

No datasets were generated or analysed during the current study.
