# Supplementary material for: Diagnostic performance of actigraphy in Alzheimer’s disease using a machine learning classifier – a cross-sectional memory clinic study
Source: Alzheimers Res Ther. 2025 May 21;17:111. doi: 10.1186/s13195-025-01751-5 (PMC12374425; doi:10.1186/s13195-025-01751-5)
Supplement: Supplementary file 1 — Supplementary Material 1 [file 13195_2025_1751_MOESM1_ESM.docx]

Supplementary Material

Related to *Diagnostic performance of actigraphy in Alzheimer’s disease using a machine learning classifier – a cross-sectional memory clinic study*

# Supplementary Tables

**Supplementary** **Table 1.** Average time spent in activity categories, steps, number of sit-to-stand movements and activity level according to diagnostic groups during daytime (6 AM-22 PM).

|  | **Alzheimer’s disease**  **(N=70)** | **Dementia with Lewy bodies (N=29)** | **Mixed AD (+CVD)**  **(N=8)** | **Vascular cognitive dysfunction (N=15)** | **Healthy control (N=48)** | **P-value** |
| --- | --- | --- | --- | --- | --- | --- |
| **Lying rest,**  minutes/day | 92.7 ± 91.2 | 149.6 ± 103.4 | 107.7 ± 112 | 146 ± 112.3 | 70.5 ± 66.2 | 0.004^1^ |
| **Lying movement,** minutes/day | 1.5 ± 1.8 | 1.5 ± 1.6 | 1.1 ± 0.9 | 0.9 ± 0.8 | 1.9 ± 1.8 | 0.120^1^ |
| **Sitting,**  minutes/day | 495.9 ± 122.7 | 480.3 ± 115.3 | 554.6 ± 178.9 | 549 ± 149.4 | 506.7 ± 92.9 | 0.331^1^ |
| **Upright standing,** minutes/day | 122 ± 53.7 | 116.8 ± 49.7 | 139.6 ± 74.3 | 89.8 ± 44.5 | 116.7 ± 35.6 | **0.106** |
| **Sporadic walking,**  minutes/day | 119.3 ± 39.9 | 96.8 ± 36.7 | 83.8 ± 35.1 | 82.5 ± 54.1 | 110 ± 34.9 | **<0.001** |
| **Walking,**  minutes/day | 118.3 ± 44 | 96.6 ± 44.7 | 67.9 ± 34.5 | 86.8 ± 46.2 | 132 ± 40.7 | **<0.001** |
| **Moderate intensity**  **(e.g., brisk walking),**  minutes/week | 47.1 ± 76.1 | 49.5 ± 75.5 | 34.2 ± 81.5 | 20.7 ± 66.3 | 99.3 ± 106.2 | **<0.001** |
| **Running,**  minutes/week | 3.9 ± 27 | 5.3 ± 19.9 | 0.4 ± 0.8 | 0.2 ± 0.4 | 4.6 ± 13.4 | **0.04** |
| **Cycling,**  minutes/day | 20.9 ± 49 | 74.2 ± 122.1 | 2.4 ± 6.7 | 14.6 ± 24.1 | 50.8 ± 76.9 | **0.002** |
| **Sit-to-stand,**  number/day | 81 ± 26.9 | 82 ± 31.5 | 62 ± 32 | 64.2 ± 17 | 83 ± 26.7 | 0.017 |
| **Steps,**  number/day | 8123 ± 3583 | 6821 ± 3509 | 4497 ± 3204 | 5518 ± 3675 | 9949 ± 4008 | **<0.001** |
| **Overall intensity,** intensity count/min | 51.6 ± 19.3 | 46.8 ± 22.8 | 30 ± 19 | 33.8 ± 20.5 | 64.1 ± 22.5 | **<0.001** |

Values are expressed as mean ± standard deviation. P-values from Kruskal-Wallis test. **Bold** indicates p-value below significance level.

**Supplementary Table 2.** Average time spent in activity categories, steps, number of sit-to-stand movements and activity level according to diagnostic groups during nighttime (22 PM – 6 AM).

|  | **Alzheimer’s disease**  **(N=70)** | **Dementia with Lewy bodies (N=29)** | **Mixed AD (+CVD)**  **(N=8)** | **Vascular cognitive dysfunction (N=15)** | **Healthy control (N=48)** | **P-value** |
| --- | --- | --- | --- | --- | --- | --- |
| **Lying rest,**  minutes/night | 448.7 ± 28.9 | 433.9 ± 26 | 431.3 ± 24.1 | 426.8 ± 35.1 | 437.4 ± 42 | **0.002** |
| **Lying movement,** minutes/night | 3.2 ± 2.1 | 3 ± 2.2 | 4.8 ± 2.8 | 3.7 ± 3.1 | 3.5 ± 1.8 | 0.132 |
| **Sitting,**  minutes/night | 18 ± 21.4 | 30.1 ± 21.5 | 29.3 ± 23.4 | 39.2 ± 36.4 | 27.2 ± 36.1 | **0.003** |
| **Upright standing,** minutes/night | 3.9 ± 5.9 | 4.4 ± 3.91 | 6.5 ± 5.2 | 3.7 ± 3.3 | 5.4 ± 10.2 | 0.220 |
| **Sporadic walking,**  minutes/night | 3.4 ± 4.2 | 4.8 ± 3.9 | 5.2 ± 3 | 3.8 ± 2.6 | 3.2 ± 3.1 | **0.042** |
| **Walking,**  minutes/night | 2.8 ± 2.9 | 4 ± 3.7 | 3 ± 1.6 | 2.9 ± 1.4 | 3.1 ± 2.4 | 0.439 |
| **Moderate intensity**  **(e.g., brisk walking),**  minutes/week | 0.2 ± 1.5 | 0 ± 0 | 0 ± 0 | 0.01 ± 0.03 | 1 ± 5.2 | 0.414 |
| **Running,**  minutes/week | 0 ± 0 | 0 ± 0 | 0 ± 0 | 0 ± 0 | 0.01 ± 0.06 | 0.637 |
| **Cycling,**  minutes/night | 0.3 ± 2.6 | 0 ± 0 | 0 ± 0 | 0 ± 0 | 0.8 ± 4.9 | 0.634 |
| **Sit-to-stand,**  number/night | 7.1 ± 4.4 | 9.4 ± 7.4 | 8.7 ± 4.1 | 7.4 ± 4.2 | 7.3 ± 3.4 | 0.709 |
| **Steps,**  number/night | 135 ± 170.2 | 181 ± 170.5 | 128 ± 96.8 | 133 ± 96.5 | 172 ± 181.9 | 0.356 |
| **Overall intensity,** intensity count/min | 3.33 ± 2.63 | 3.94 ± 2.89 | 4.13 ± 1.16 | 3.80 ± 1.87 | 3.90 ± 2.50 | 0.144 |

Values are expressed as mean ± standard deviation. P-values from Kruskal-Wallis test. **Bold** indicates p-value below significance level.

**Supplementary Table 3**. Performance in differentiating AD vs. other dementia etiologies in a leave-one-out scheme using either logistic regression (machine learning) or thresholding of average intensity count during the day. The 95% confidence intervals were estimated using the Clopper–Pearson's method.

|  |  |  | **Sensitivity** | **Specificity** | **Accuracy** | **Precision** | **F1** |
| --- | --- | --- | --- | --- | --- | --- | --- |
| Disease groups vs. Healthy | **Logistic regression (full feature set)** | Default (50 % probability) | 67.2  (59.4 - 74.1) | 68.8  (61.3 - 75.7) | 67.6  (60.1 - 74.6) | 84.5  (78.4 - 89.8) | 74.9  (67.5 - 81.0) |
|  |  | Optimized Threshold | 95.1  (90.9 - 97.9) | 25.0  (19.0 - 32.5) | 75.3  (68.1 - 81.6) | 76.3  (69.4 - 82.6) | 84.7  (78.4 - 89.8) |
|  | **Single feature** | Default | 84.4  (78.4 - 89.8) | 25.0  (19.0 - 32.5) | 67.6  (60.1 - 74.6) | 74.1  (66.9 - 80.5) | 78.9  (71.9 - 84.7) |
|  |  | Optimized Threshold | 86.1  (79.7 - 90.7) | 43.8  (36.0 - 51.3) | 74.1  (66.9 - 80.5) | 79.5  (72.5 - 85.2) | 82.7  (76.4 - 88.3) |
| AD vs. healthy | **Logistic regression (full feature set)** | Default | 57.1  (47.3 - 65.9) | 64.6  (55.1 - 73.0) | 60.2  (50.7 - 69.1) | 70.2  (61.2 - 78.4) | 63.0  (53.3 - 71.4) |
|  |  | Optimized Threshold | 85.7  (77.9 - 91.4) | 37.5  (28.6 - 46.7) | 66.1  (56.8 - 74.6) | 66.7  (57.7 - 75.3) | 75.0  (66.6 - 82.9) |
|  | **Single feature** | Default | 80.0  (71.3 - 86.5) | 31.3  (23.1 - 40.5) | 60.2 (50.7 - 69.1) | 62.9 (53.3 - 71.4) | 70.4 (61.2 - 78.4) |
|  |  | Optimized Threshold | 85.7 (77.9 - 91.4) | 43.8 (34.9 - 53.5) | 68.6 (59.5 - 76.9) | 69.0 (59.5 - 76.9) | 76.4 (67.6 - 83.6) |

**Supplementary Table 4**. Performance in differentiating AD vs. other dementia etiologies in a leave-one-out scheme using either logistic regression (machine learning) or thresholding of average intensity count during the day. The 95% confidence intervals were estimated using the Clopper–Pearson's method.

|  |  |  | **Sensitivity** | **Specificity** | **Accuracy** | **Precision** | **F1** |
| --- | --- | --- | --- | --- | --- | --- | --- |
| AD vs. DLB | **Logistic regression (full feature set)** | Default (50 % probability) | 75.9  (66.1 - 83.8) | 78.6  (69.4 - 86.4) | 77.8  (68.3 - 85.5) | 59.5  (49.3 - 69.3) | 66.7  (56.5 - 75.8) |
|  |  | Optimized Threshold | 62.1  (51.3 - 71.2) | 88.6  (81.0 - 94.3) | 80.8  (71.7 - 88.0) | 69.2  (59.6 - 78.5) | 65.5  (55.4 - 74.9) |
|  | **Single feature** | Default | 10.3  (5.0 - 17.8) | 94.3  (87.3 - 97.7) | 69.7  (59.6 - 78.5) | 42.9  (32.5 - 52.8) | 16.7  (9.5 - 24.9) |
|  |  | Optimized Threshold | 24.1  (16.2 - 33.9) | 94.3  (87.3 - 97.7) | 73.7  (63.9 - 82.1) | 63.6  (53.4 - 73.1) | 35.0  (26.0 - 45.6) |
| AD vs. CVD | **Logistic regression (full feature set)** | Default | 78.3  (68.8 - 86.3) | 75.7  (65.2 - 83.6) | 76.3  (66.4 - 84.5) | 51.4  (41.0 - 62.1) | 62.1  (51.7 - 72.2) |
|  |  | Optimized Threshold | 69.6  (59.5 - 79.0) | 95.7  (89.4 - 98.8) | 89.2  (81.1 - 94.7) | 84.2  (74.8 - 90.7) | 76.2  (66.4 - 84.5) |
|  |  | Default | 34.8  (24.9 - 45.0) | 94.3  (87.9 - 98.2) | 79.6  (69.9 - 87.2) | 66.7  (56.1 - 76.1) | 45.7  (35.8 - 56.9) |
|  | **Single feature** | Optimized Threshold | 47.8  (36.9 - 57.9) | 94.3  (87.9 - 98.2) | 82.8  (73.6 - 89.8) | 73.3  (62.9 - 81.8) | 57.9  (47.4 - 68.2) |

**Supplementary Table 5.** Output probability of our ML model for the control group.

| Variable | Estimate | Standard error | t-statistic | P-value |
| --- | --- | --- | --- | --- |
| (Intercept) | -0,1898 | 0.47 | -0.403 | 0.69 |
| Age | 0.007 | 0.0061 | 1.17 | 0.25 |
| Sex | 0.023 | 0.096 | 0.24 | 0.81 |
| Educational level | 0.0036 | 0.018 | 0.2 | 0.84 |
| Antidepressant medication | -0.086 | 0.18 | -0.49 | 0.63 |


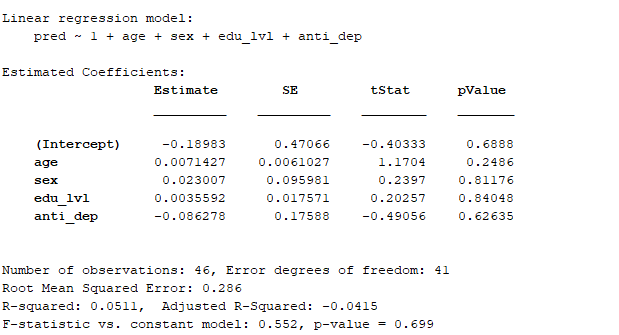


**Supplementary Table 6.** Output probability of our ML model for the dementia group.

| Variable | Estimate | Standard error | t-statistic | P-value |
| --- | --- | --- | --- | --- |
| (Intercept) | -0.25 | 0.32 | -0.79 | 0.43 |
| Age | 0.012 | 0.00384 | 3.14 | **0.0022** |
| Sex | 0.0065 | 0.053 | 0.123 | 0.9 |
| Educational level | -0.084 | 0.0068 | -1-124 | 0.22 |
| Antidementia medication | -0.074 | 0.056 | -1.34 | 0.183 |
| Antidepressant medication | 0.097 | 0.079 | 1.23 | 0.22 |
| Antipsychotic medication | 0.0345 | 0.158 | 0.218 | 0.83 |
| Hypnotic medication | 0.386 | 0.0272 | 1.42 | 0.16 |
| Sedative/analgesic medication | 0.33 | 0.16 | 1.01 | 0.31 |
| AD | 0.068 | 0.067 | 1.01 | 0.31 |
| DLB | 0.144 | 0.081 | 1.78 | 0.077 |
| Cerebrovascular disease | 0.23 | 0.1 | 2.23 | **0.028** |


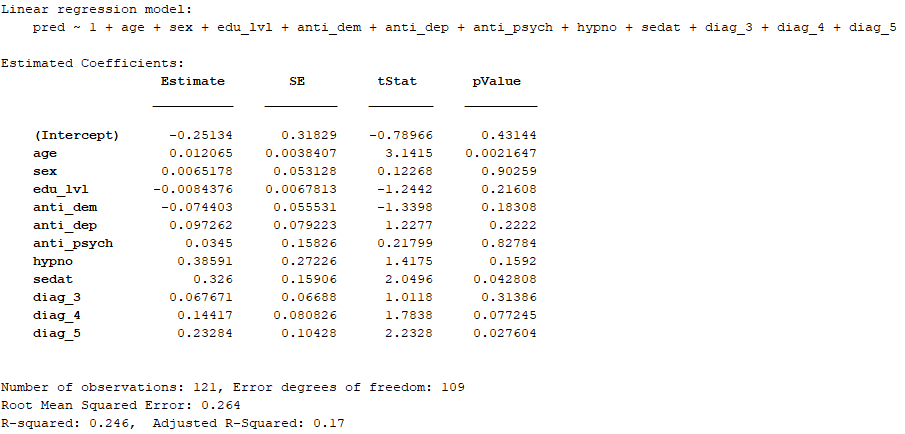


**Supplementary Figure 1.** Performance in differentiating groups in a leave-one-out scheme using either logistic regression (Machine learning) or thresholding of average intensity count during the day (Single feature).


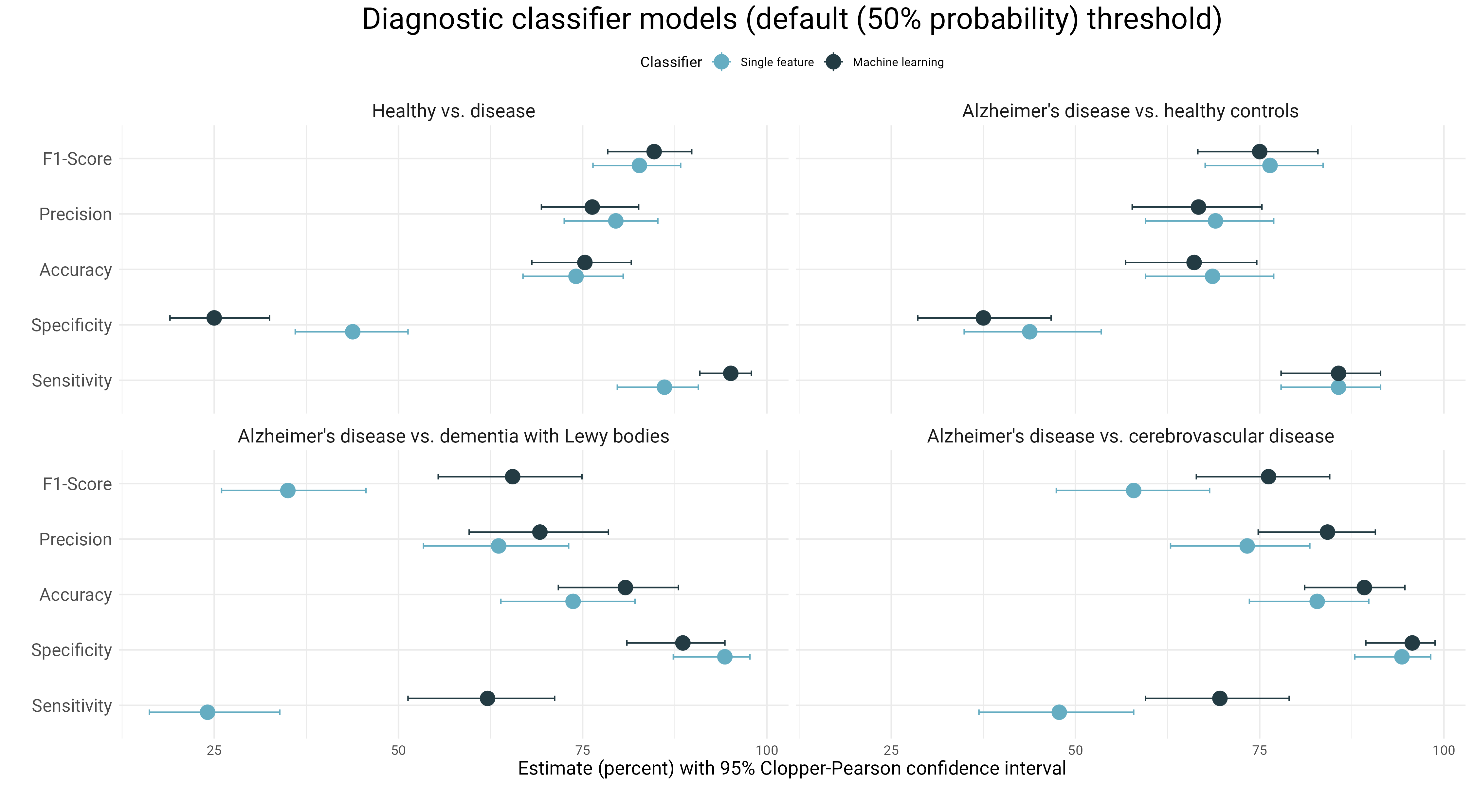


**Supplementary Figure 2.** Feature importance is shown for machine learning models. The Y-axis is the average absolute value for the logistic regression coefficients. A complete description of variable names is found below.


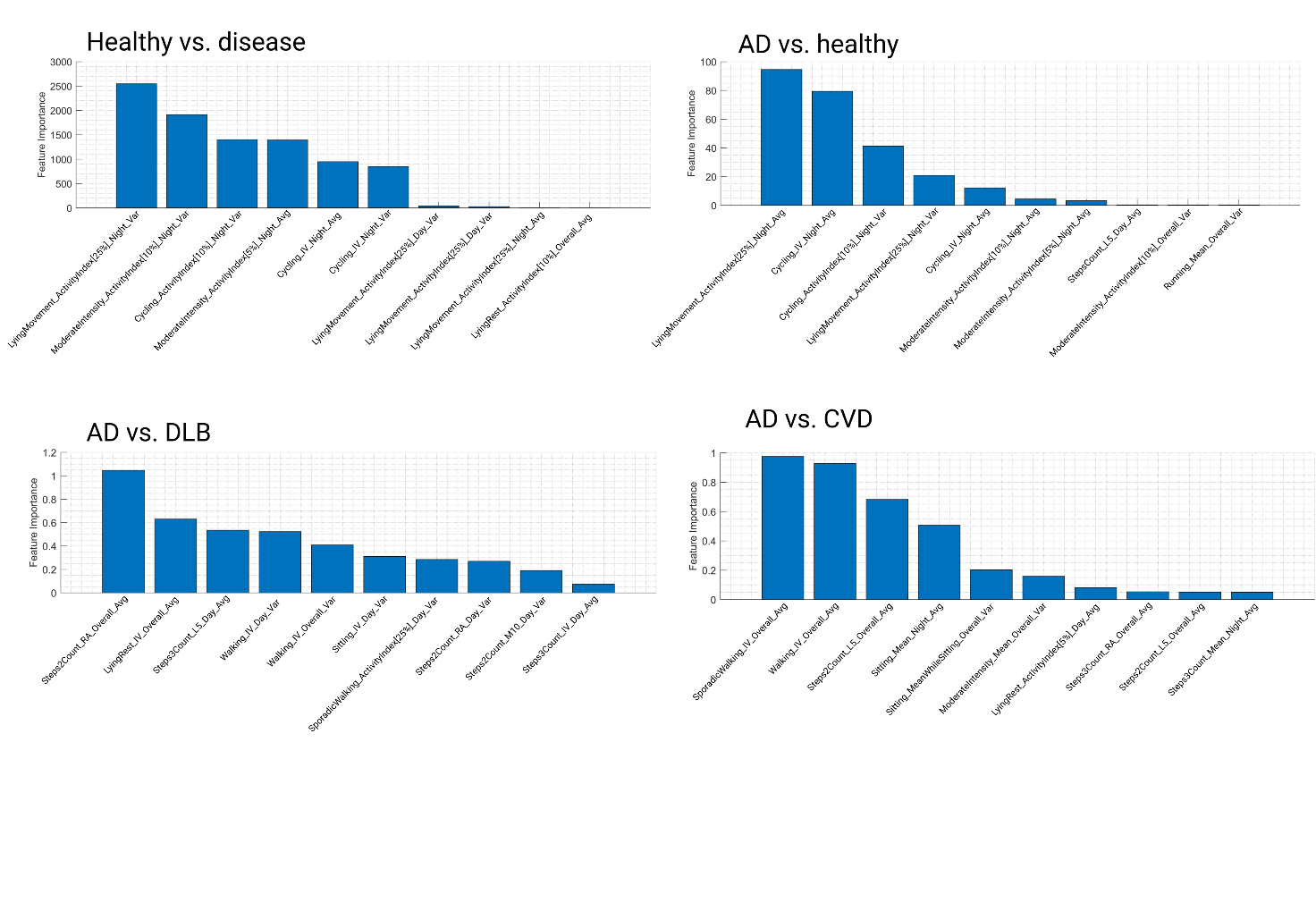


# **Variable description**

Feature names are structured as follows:

[activity type / acitivity count] _ [feature] _ [period] _ [per 24-hour period summary]

[activity type / acitivity count] can be {LyingRest, LyingMovement, UprightStanding, SporadicWalking, Walking, ModerateIntensity, Running, Cycling, Sitting, IntensityCount, Steps1Count, Steps2Count, Steps3Count, Sit2StandCount}

[feature] can be {Mean, IV, L5, M10, TimingL5, TimingM10, RA, MeanWhileSitting, ActivityIndex[X%]}

[period] can be {Overall, Day, Night}

[per 24-hour period summary] can be {Avg, Var}

The MeanWhileSitting feature is calculated based on the 15-minutes epochs where sitting is the dominant activity (>50 %).

# **SENS Motion Actigraph Dual Sensor System**

The SENS actigraph system takes raw accelerometric data and applies a proprietary algorithm that generates activity categories accordingly (Bartholdy et al., 2018):

No data; No data collected.

Lying rest: The participant lies down with little or no movement.

Sitting rest; The participant sits with little or no movement.

Lying movement; Movements of the participant in a lying state.

Sitting movement; Movements of the participant in a sitting state.

Standing; The participant stands upright.

Walking; The participant walks continuously.

Sporadic walking; The participant stands upright, but with a few movements. The activity relates to 'Standing' and 'Walking', but it is a middle ground and irregular.

Cycling & similar; The participant cycles.

Moderate intensity; The participant is in a brisk walk, where the activity is higher than 'Walking', but lower than 'High intensity'.

High intensity; The participant runs.

Number of position changes (sitting to standing)

Steps taken (3 step categories, explained further below)

Data is analyzed in 5-second epochs, and each epoch is estimated to be belonging to a certain activity category. The categories are characterized by (paraphrased from the SENS Motion Actigraph System Manual for Researchers) :

**Intensity count:** The activity intensity is calculated as the average magnitude of movement based on high pass filtered 3 axis accelerometer measurements at 12 Hz sampling frequency, subtracting the noise present on each measurement axis, done on each 5-second epoch. This measurement gives a good indication of the intensity of the measurement. The intensity count in a 5-second interval is normally between 0-100, where 100 is high intensity.

**Resting:** This category is estimated when the sensor is in a horizontal position (meaning that the leg is in a horizontal position) (+- about 45 degrees). At higher intensities either the “laying or sitting movement” category is estimated or there is a chance that the patient is actually biking, and signals are further analyzed to check this (see biking). This category should be seen as an indication of the patient being sedentary, sitting or lying down.

**Sitting:** This category is estimated when the thigh mounted sensor is in a horizontal position (meaning that the leg is in a horizontal position) while the upper body mounted sensor is mostly in a vertical position (meaning that the upper body is upright) (+- about 45 degrees). At higher intensities there is a chance that the patient is actually biking, and signals are further analyzed to check this (see biking). This category should be seen as an indication of the patient being sedentary, sitting down.

**Standing:** This category is estimated when the sensor is in a vertical position (meaning that the leg is in a vertical position - standing) (+- about 45 degrees) and the intensity readings are lower than a minimum movement threshold of about 0.1 G. This category should be seen as an indication of the patient standing still with only very minor insignificant movements recognized.

**Biking:** The biking category is estimated if a repeating pattern is recognized with a frequency above 0.2 Hz, where both legs move all the time, and the measured movements are symmetrical. This is recognized based on pattern recognition. Also, the intensity must be above the measurement threshold. This category should be seen as an indication of the patient doing a cyclic leg movement for more than 1-2 minutes (with pauses). Smaller biking intervals (trips) below 1 minute are not seen often and will likely be detected as walking / sporadic walking.

**Sporadic walking**: All movements in upright (vertical) position where the vector magnitude is above the movement threshold, but below the moderate intensity, and where the movement is not recognized as falling into other categories. This will include epochs where an activity is starting towards the end of the epoch or walking just a few steps before again standing still. Also biking very slowly for shorter periods will fall into this category.

**Walking:** The walking category is estimated if a repeating pattern with an intensity above the measurement threshold, but below the threshold for moderate activity as well as a frequency above 0.2 Hz is recognized based on pattern recognition. As one leg at a time takes a step forward, and as the sensor is mounted on one leg, the measured movement must be asymmetrical, and thus the pattern recognition does not yield biking. This category should be seen as an indication of the patient walking for a period of 5-10 seconds continuously. Smaller walking intervals (trips) below 3-9 seconds will likely be detected as sporadic walking.

**Moderate Intensity:** This training category is used for any walking activity above the walking intensity interval for a period of at least 3-9 seconds continuously (which is not recognized as biking). This category can also be defined as "brisk walking," where the intensity is higher than regular walking, but lower than high intensity activities such as running.

**High Intensity/Running:** The high intensity category is used for any walking activity above the moderate intensity interval for a period of 3-9 seconds continuously (which is not recognized as biking). This category should be seen as an indication of the patient running or doing high intensity training involving their legs. Smaller running intervals (trips) below 3-9 seconds will likely be detected as moderate intensity or walking.

**Steps taken:** During sporadic walk, walking or training the number of steps is recognized.

Steps taken during continuous walking activity and training are based on an analysis in the frequency domain. The characteristic frequency of the walking motion is recognized during a 5 second interval, providing a step count. This category of steps is summarized as “*Step 1*”. This is the category recognized as “Steps” in the present study.

Steps taken during sporadic walking where no continuous frequency can be recognized in the 5 second interval are summarized as 2 steps per 5 second sporadic walking interval. This is summarized as “*Step 2*”.

Steps taken during low intensity walking where a continuous frequency can be recognized in the 5 second interval are based on an analysis in the frequency domain. The characteristic frequency of the walking motion is recognized during a 5 second interval, providing a step count. This category of steps is summarized as “*Step 3*”.

**Laying or sitting movement:** All movements in sitting / laying (horizontal) position, where the vector magnitude is above the movement threshold, where the movement is not recognized as falling into other categories. Ex. biking very slowly with the sensor in a mostly horizontal angle for shorter periods will fall into this category.

**Standing movement (not walking):** All movements in upright (vertical) position where the vector magnitude is above the movement threshold, though being very small / slow movements below the moderate intensity, and where the movement is not recognized as falling into other categories. Ex. biking very slowly with the sensor in a mostly vertical angle for shorter periods will fall into this category.

**References**

Bartholdy, C., Gudbergsen, H., Bliddal, H., Kjærgaard, M., Lykkegaard, K. L., & Henriksen, M. (2018). Reliability and Construct Validity of the SENS Motion® Activity Measurement System as a Tool to Detect Sedentary Behaviour in Patients with Knee Osteoarthritis. *Arthritis*, *2018*, 1–9. https://doi.org/10.1155/2018/6596278
